# Meta-analysis of the effects of ischemic postconditioning on structural pathology in ST-segment elevation acute myocardial infarction

**DOI:** 10.18632/oncotarget.23450

**Published:** 2017-12-16

**Authors:** Baohui Lou, Yadong Cui, Haiyang Gao, Min Chen

**Affiliations:** ^1^ Department of Radiology, Beijing Hospital, National Center of Gerontology, Beijing, China; ^2^ Department of Cardiology, Beijing Hospital, National Center of Gerontology, Beijing, China; ^3^ Graduate School of Peking Union Medical College, Beijing, China

**Keywords:** local ischemic postconditioning, remote ischemic postconditioning, acute myocardial infarction, cardiac magnetic resonance imaging, structural effect

## Abstract

In this meta-analysis, we assessed cardiac magnetic resonance imaging data to determine the effects of local and remote ischemic postconditioning (LPoC and RPoC, respectively) on structural pathology in ST-segmentel elevation acute myocardial infarction (STEMI). We searched the Pubmed, Embase and Cochrane Library databases up to May 2017 and included 12 randomized controlled trials (10 LPoC and 2 RPoC)containing 1069 study subjects with thrombolysis in myocardial infarction flow grade 0~1. Weighed mean difference (WMD), standardized mean difference (SMD), and odds ratio (OR) were used for the pooled analysis. Random-effect model was used for the potential clinical inconsistency. LPoC and RPoC increased the myocardial salvage index (*n* = 5; weighted mean difference (WMD) = 5.52; *P* = 0.005; I^2^ = 76.0%), and decreased myocardial edema (*n* = 7; WMD = −3.35; *P* = 0.0009; I^2^ = 18.0%). However, LPoC and RPoC did not reduce the final infarct size (*n* = 10; WMD = −1.01; *P* > 0.05; I^2^ = 68.0%), left ventricular volume (*n* = 10; standardized mean difference = 0.23; *P* > 0.05; I^2^ = 93.0%), the incidence of microvascular obstruction (*n* = 6; OR = 0.99; *P* > 0.05; I^2^ = 0.0%) or the extent of microvascular obstruction (*n* = 3; WMD = −0.09; *P* > 0.05; I^2^ = 6.0%). This meta-analysis shows that LPoC and/or RPoC improves myocardial salvage and decreases myocardial edema in STEMI patients without affecting final infarct size, left ventricular volume or microvascular obstruction.

## INTRODUCTION

Timely restoration of coronary perfusion is the most effective strategy to limit infarction size (IS) and improve clinical outcomesin patients with ST-segment elevation acute myocardial infarction (STEMI) [1]. However, the progressive changes in structure and morphology of the left ventricle after ischemic myocardial reperfusion is associated with 25% of heart failure (HF) cases [2, 3]. Hence, accurate evaluation of the effects of STEMI therapy on cardiac structural pathology is critical [4].

Ischemic postconditioning (PoC) by brief, repetitive cycles of ischemia and reperfusion in the heart (local postconditioning, LPoC) [5, 6] or limbs (remote postconditioning, RPoC) [7, 8] during early reperfusion has been shown to reduce ischemia [9]. The effect of ischemic postconditioning on cardiac enzyme levels and left ventricular function have been confirmed in the clinical trials of acute myocardial infarction (AMI) [10–12]. However, the findings on the effects of ischemic postconditioning on structural pathologyof STEMI because of the variety of imaging techniquesused such as angiography [13], echocardiography [14, 15], and single-photonemission computed tomography (SPECT) [16].

Cardiac magnetic resonance imaging (cMRI) has emerged as the most accurate and reliable tool for the evaluation of cardiac structure. Moreover, contrast-enhanced cMRI has been widely used to measure the infarct size with high spatial resolution [17–19]. In addition toventricular dimensions and infarct size, cMRI simultaneously measures myocardial salvage index (MSI), microvascular obstruction (MVO), and myocardial edema todetermine area at risk (AAR) inhigh-quality cross-sectionalimages, therebyenabling accurate anatomicaldelineation [20]. cMRI detection has also been employed in clinical trials to determine the clinical utility of PoC in STEMI [21–24]. Therefore, we conducted a comprehensive meta-analysis of clinical trials that have used cMRI to identify the potential benefits of LPoC and RPoC in STEMI patients.

## RESULTS

### Study selection strategy

**Figure 1:**
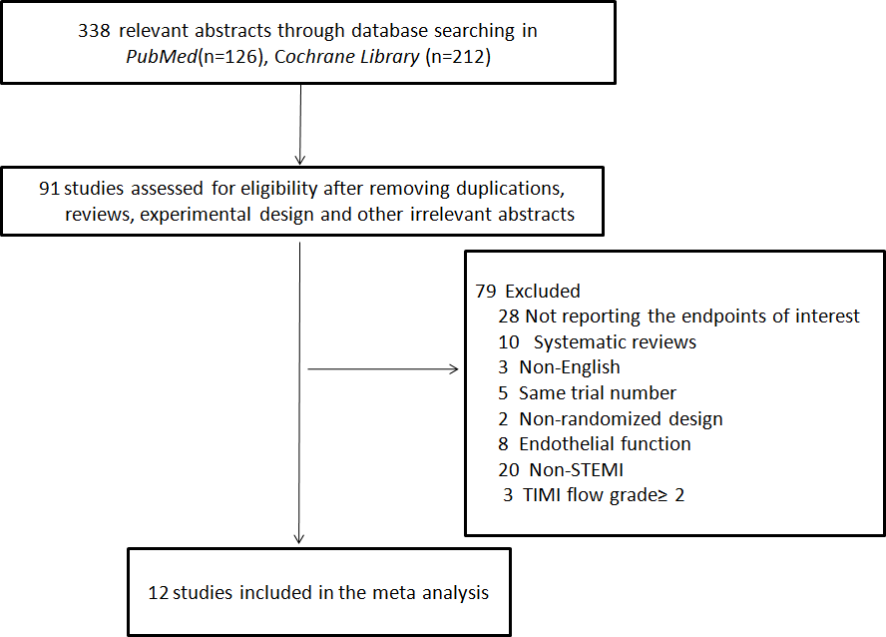
Flow chart of the literature search and selection strategy of eligible studies The flow chart shows search and selection of cMRI imaging studies on the status of structural pathology of ST-segmental elevation myocardial infarction (STEMI) patients with thrombolysis in myocardial infarction (TIMI)grade 0~1 that underwent percutaneous coronary intervention (PCI).

We searched Pubmed, MEDLINE and Cochrane library databases and identified 338potential studies after excluding articles that were duplications, reviews, experimental designs, and other irrelevant contents (Figure 1). We further excluded 79 studies that were systematic reviews (*n* = 10), non-English reports (*n* = 3), had same trial numbers (*n* = 5), were non-RCT (*n* = 2), and endothelial trials (*n* = 8), studied STEMI patients with Thrombolysis in myocardial infarction (TIMI) flow grade ≥ 2 (*n* = 3), and non-STEMI patients (*n* = 20) and for not reporting the primary endpoints of interest (*n* = 28). Finally, we included 12 trials [13, 14, 21–30]with 1069STEMI patients(LPoC, (*n* = 920; RPoC, (*n* = 149)undergoing cMRI assessment after percutaneous coronary intervention (PCI) (Figure 1).

### Study characteristics

The study design and patient characteristics are summarized in Tables 1 and 2. The LPoC protocol [cycles ×ischemia/reperfusion (I/R)] was 4 × 60 s/60 s in eight studies [13, 14, 22, 25, 26, 28–30], and 4 × 30 s/30 s in two studies [21, 27]. The RPoC protocol (cycles× I/R) was 3 × 5 min/5 min in both studies that reported it [23, 24]. The symptom-to-balloon time was 2.92-5.47 h, and the TIMI flow grade was 0~1. The patients were followed up for 12 months. Final infarct size (IS) was reported in 10LPoC-related [13, 14, 21–27, 30] and twoRPoC-related [23, 24] studies. Myocardial salvage index (MSI) was reported in fiveLPoC-related [22, 27, 28, 30] and 1 RPoC-related [24] studies. Myocardial edema was reported in seven LPoC-related [21, 22, 26, 27, 30] and two RPoC-related [23, 24] studies. Left ventricular (LV) volume parameters were reportedin ten LPoC-related [13, 14, 22, 25–27, 29, 30] and two RPoC-related [23, 24] studies. Microvascular obstruction (MVO) was reported in seven LPoC-related [13, 14, 22, 25, 30] and RPoC-related [23, 24] studies. The Jadad score for ten studies [13, 14, 21–24, 26–28, 30] was ≥ 3 and 2 for two other studies [25, 29].

**Table 1:** Summarized study design of the included randomized trials

Study	Country	AMI	TIMI flow grade	Protocol Algorithm	Conditioning Delay(s)	Pts. No.	Clinical Endpoints	Symptom- to-balloon (h)	Cardiac Imaging	Follow-up	Jadad score
						PoC vs Ctrl					
LPoC											
Lonborg 2010^[21]^	Denmark	STEMI	0~1	30s^4	< 60 s	43 vs 43	IS, ME	4.16	CMR	3 mons	3
Sörensson 2010^[25]^	Sweden	STEMI	0	60s^4	60 s	33 vs 35	IS, MVO, LVEDVI	2.92	CMR	12 mons	2
Frexia 2012^[13]^	Spain	STEMI	0~1	60s^4	60s	31 vs 31	IS, MVO. LVEDV	5.47	CMR	6 mons	5
Taraniti 2012^[14]^	Italy	STEMI	0~1	60s^4	< 60 s	37 vs 38	IS, MVO, LVEDVI	3.38	CMR	1 mon	3
Thuny 2012^[26]^	France	STEMI	0~1	60s^4	< 60 s	25 vs 25	IS, ME, LVEDVI	4.20	CMR	4 days	3
Dwyer 2013^[27]^	Canada	STEMI	0~1	30s^4	< 60 s	39 vs 40	MSI, ME, LVEDVI	2.66	CMR	5 days	3
Limalanathan 2013^[28]^	Norway	STEMI	0~1	60s^4	60 s	120 vs 129	MSI	3.00	CMR	4 mons	3
Elzbieciak 2013^[29]^	Poland	STEMI	0~1	60s^4	< 60 s	18 vs 21	LVEDV	4.59	CMR	3 mons	2
Kim 2015^[22]^	Korea	STEMI	0~1	60s^4	< 60 s	56 vs 55	IS, MSI, MVO, ME, LVEDVI	4.77	CMR	3 days	3
Bodi 2014^[30]^	Spain	STEMI	0~1	60s^4	60 s	49 vs 52	IS, MSI, MVO, ME, LVEDVI	3.20	CMR	6 days	3
RPoC											
Crimi 2013^[23]^	Italy	STEMI	0~1	3 × 5 min/5 min at lower limb (200 mmHg)	Immediately	30 vs 36	IS, MVO, ME	3.01	CMR	4 mons	5
White 2014^[24]^	UK	STEMI	0~1	3 × 5 min/5 min at upper limb (200 mmHg)	Immediately	43 vs 40	IS, MSI, MVO, ME	3.10	CMR	6 day	5

Note: AMI, acute myocardial infarction; STEMI, ST-segmental elevation myocardial infarction; TIMI, Thrombolysis In Myocardial Infarction ;Pts. No., patient number; IS, infarct size; MSI, myocardial salvage index; MVO, microvascular obstruction; ME, myocardial edema; LVEDV, left ventricular end-diastolic volume; LVEDVI, indexed LVEDV; CMR, cardiac magnetic resonance imaging; N.A, not available; LPoC, local ischemic postconditioning; RPoC, remote ischemic postconditioning; Ctrl, control.

**Table 2:** Summarized patient characteristics of the included randomized trials

Study	Age	Male(%)	DM(%)	HP(%)	Smk (%)	DysLip(%)	Muti- vessel(%)	LAD(%)	Direct Stent(%)	β-blocker(%)	Statins(%)	GP(%)
**LPoC**												
Lonborg 2010 ^[21]^	61.5	78.0	6.9	34.8	55.1	43.2	19.5	42.0	0.0	19.5	11.9	83.1
Sörensson 2010 ^[25]^	62.5	32.5	14.5	22.4	27.6	63.2	36.8	37.0	2.6	6.9	8.3	79.0
Frexia 2012 ^[13]^	59.5	77.9	20.0	49.5	56.6	39.4	N.A	30.0	58.0	26	19	N.A
Taraniti 2012 ^[14]^	59.6	84.6	10.3	53.9	71.8	50.0	5.1	42.0	100.0	27.0	11.5	98.7
Thuny 2012 ^[26]^	57.0	74.0	17.0	44.0	66.0	N.A	N.A	56.0	100.0	N.A	N.A	74.0
Dwyer 2013 ^[27]^	57.0	88.2	9.8	37.3	44.0	N.A	N.A	48.0	0.0	N.A	N.A	83.3
Limalanathan 2013 ^[28]^	60.0	82.0	3.0	26.9	51.1	N.A	33.1	48.0	0.0	N.A	N.A	N.A
Elzbieciak 2013 ^[29]^	59.2	76.9	23.1	84.6	59.0	74.4	48.7	100.0	0.0	N.A	N.A	N.A
Kim 2015 ^[22]^	60.0	76.7	24.4	45.7	52.3	42.6	N.A	46.0	13.4	100.0	100.0	N.A
Bodi 2014 ^[30]^	60.0	83.0	27.0	51.0	59.0	53.0	38.0	N.A	N.A	80.0	89.0	60.0
**RPoC**												
Crimi 2013 ^[23]^	58.5	87.5	12.0	53.5	53.5	31.5	35.0	N.A	N.A	100	100	95.5
White 2014 ^[24]^	58.4	33.6	2.93	9.93	20.5	11.4	N.A	N.A	N.A	N.A	N.A	N.A

Note: DM, diabetes mellitus; HP, hypertension; Smk, smoking; DysLip, dyslipidemia; LAD, left anterior descending artery; GP, glycoprotein IIb/IIIa inhibitor ; N.A, not available; LPoC, local ischemic postconditioning; RPoC, remote ischemic postconditioning; Ctrl, control.

### Effects of ischemic post conditioning on final IS, MSI, and myocardial edema

**Figure 2:**
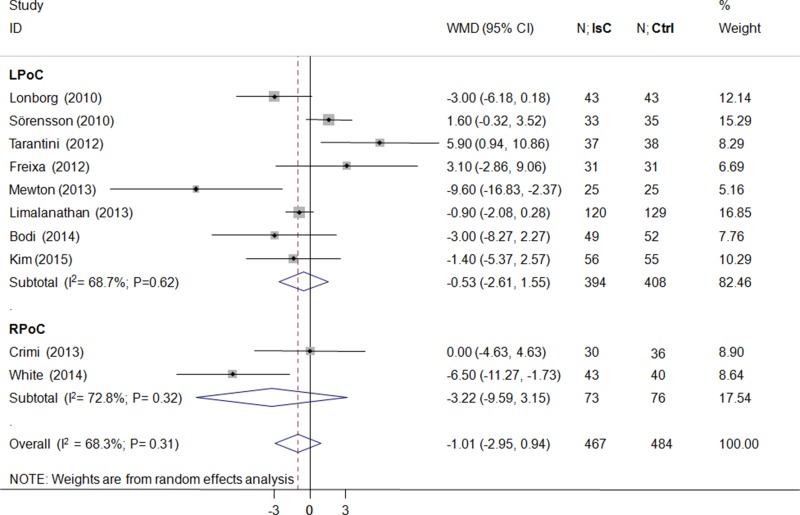
Effects of local and remote ischemic postcondtioning on final infarction size Histogram plots showing final infarction sizes(IS; percentage of left ventricle) in STEMI patients that underwent LPoC and RPoC relative to controls. As shown, ischemic postconditioning (PoC) did not improve IS(weighted mean difference(WMD) = −1.01; *P* = 0.31). Note: LPoC, local ischemic postconditioning; RPoC, remote ischemic postconditioning; PoC, ischemic postconditioning ; Ctrl, control.

**Figure 3:**
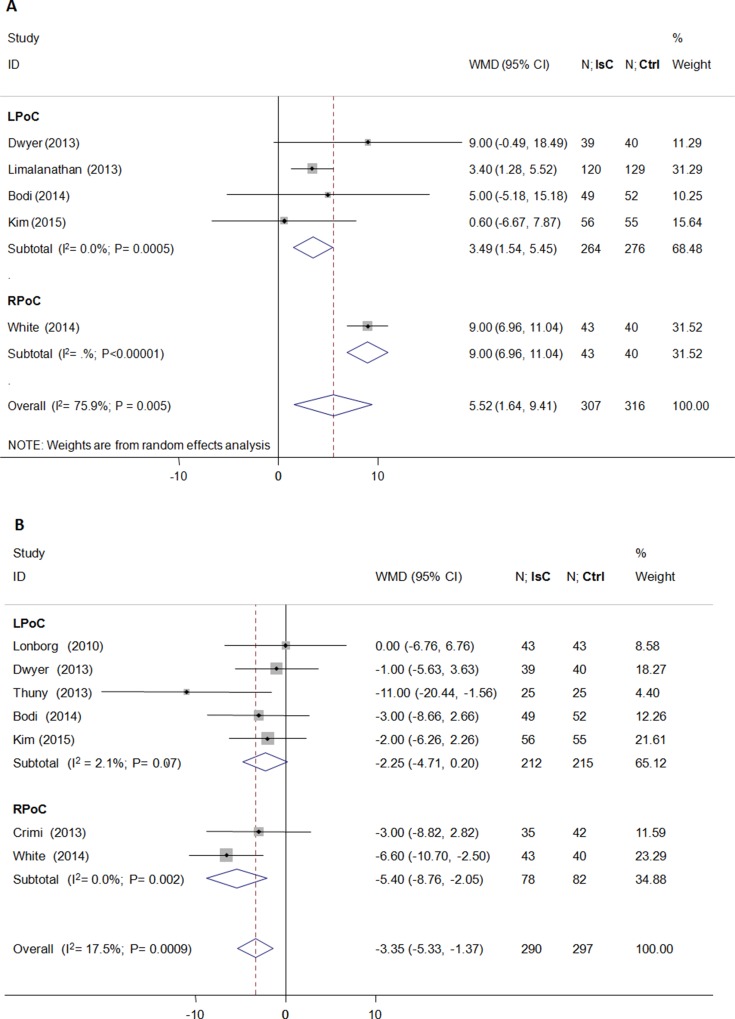
Effects of local and remote ischemic postcondtioning on myocardial salvage and edema Histogram plots showing that LPoC and RPoC increased (**A**) myocardial salvage (percentage of left ventricle; WMD = 5.52; *P* = 0.005) and reduced (**B**) myocardial edema(percentage of left ventricle; WMD = −3.35; *P* = 0.0009) in STEMI patients that underwent LPoC and RPoC relative to controls.

As shown in Figure 2, the final IS(%) was not significantly reduced by PoC (weighted mean difference [WMD] = −1.01; 95% CI: −2.95 to 0.94; *P* > 0.05) and demonstrated heterogeneity (*I*^2^ = 68.0%). Both LPoC and RPoC increased the potential of MSI(%)(WMD = 5.52; 95% CI: 1.64 to 9.41; *P* = 0.005; *I*^2^ = 76.0%; Figure 3A). Patients treated by LPoC (WMD = −2.25; 95% CI: −4.71 to 0.20; *P* = 0.07; *I*^2^ = 2.0%), or RPoC (WMD = −5.40; 95% CI: −8.76 to −2.05; *P* = 0.002; *I*^2^ = 0.0%) showed decreased percent myocardial edema (Figure 3B).

### Effect of ischemic postconditioning on LV volume and microvascular obstruction

**Figure 4:**
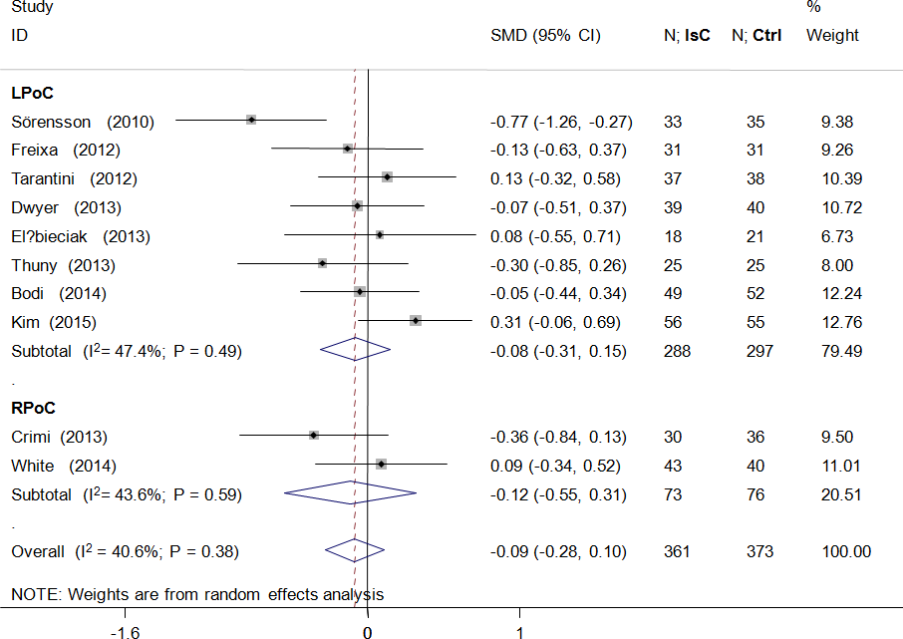
Effects of local and remote ischemic postcondtioning on left ventricular volume Histogram plots show that LPoC and RPoC did not reduce left ventricular volume (standardized mean difference(SMD) = −0.09; *P* = 0.38) in STEMI patients that underwent LPoC and RPoC relative to controls.

**Figure 5:**
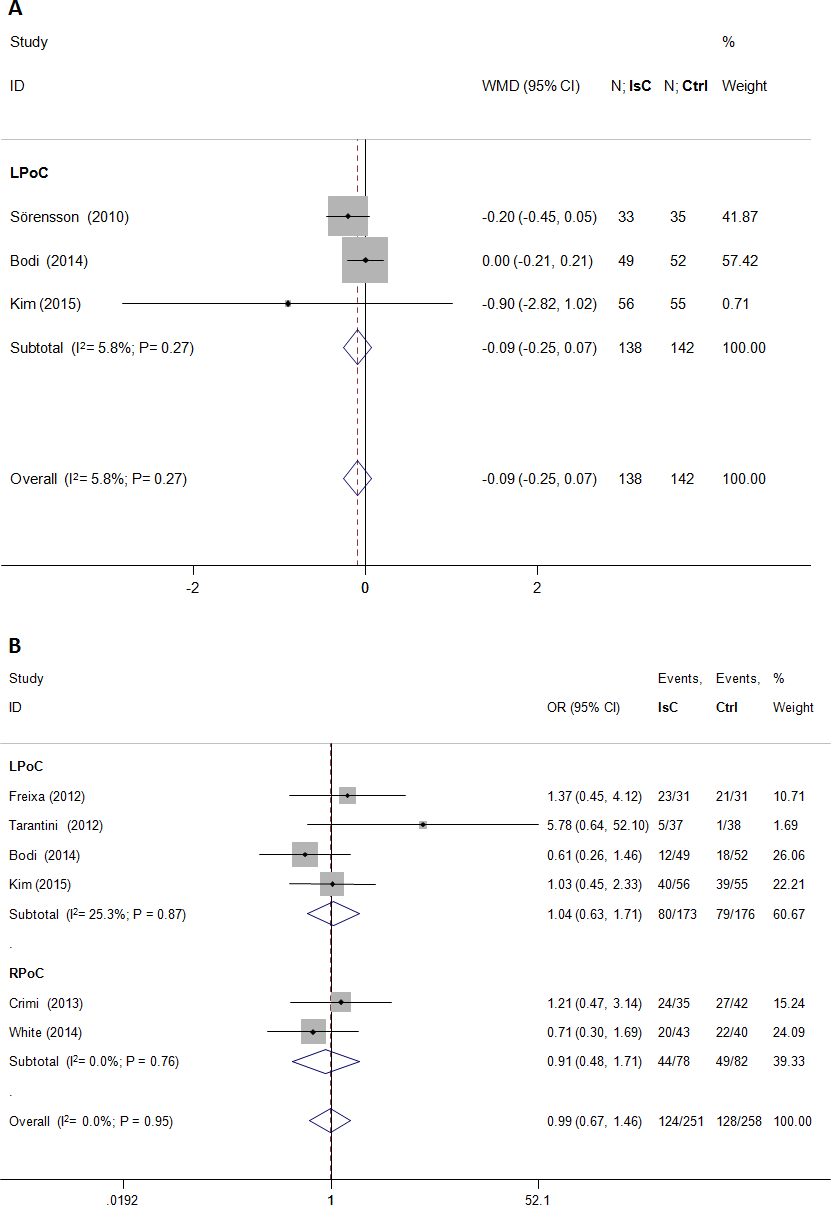
Effects of local and remote ischemic postcondtioning on microvascular obstruction Histogram plots show that LPoC and RPoC did not reduce (**A**) extent of microvascular obstruction (WMD = −0.09; *P* = 0.27) and (**B**) the incidence of microvascular obstruction (odds ratio (OR) = 0.99; *P* = 0.95].

PoC did not attenuate left ventricular volume after PCI (standardized mean difference [SMD] = −0.09; 95% CI: −0.28 to 0.10; *P* > 0.05) and showed significant heterogeneity (*I*^2^ = 40.6%; Figure 4). Microvascular obstruction(MVO) was reported in 509 (49.5%) study subjects. PoC did not reduce the risk of MVO(OR = 0.99; 95% CI: 0.67 to 1.46; *P* > 0.05; *I*^2^ = 0.0%; Figure 5A), or the extent of MVO(%) (WMD = −0.09, 95% CI: −0.25 to 0.07; *P* > 0.05; *I*^2^ = 6.0%; Figure 5B).

## DISCUSSION

In this meta-analysis of 12 randomized trials, weassessed1069 STEMI patients that underwent PCI by cardiac magnetic resonance imaging(cMRI). We observed that both LPoC and/or RPoC reduced the extent of MSI and myocardial edema, thereby offering cardioprotection. However, LPoC and RPoC did not affect final IS, LV volume, and the incidence or the extent of MVO. This meta-analysis is the first comprehensive analysis to evaluate structural effects of ischemic postconditioning in STEMI patients using cMRI.

The protective potential of ischemic postconditioning (PoC) for STEMI patients has been confirmed in clinical trials by assessing cardiac enzyme levels and left ventricular function [11, 12] and systematically reviewed previously [10]. Some trials have explored the structural effects of PoC in STEMI by angiography [13], echocardiography [14, 15] and SPECT [16]. In order to increase the consistency, we included studies that reported structural effects of PoC in STEMI as assessed by cMRI, which accurately measures the infarct size and LV volumes [17–19]. Thus, our meta-analysis provides more solid evidence about the structural effects of PoC in STEMI.

During ischemia/reperfusion (I/R), the hydrostatic pressure within interstitial space increases and results in myocardial edema. This contributes to capillary compression and aggravates the extent of cell damage, which is characteristic of severe I/R injury. Since myocardial edema is central to I/R injury, it is critical to analyze the positive effects of ischemic postconditioning. In a dog model, *ex vivo* assessment of water content showed that LPoC reduced myocardial edema [31]. Improved detection of *in vivo* myocardial edema by non-invasive T2-weighted imaging [32, 33] has led to evaluation of the efficacy of ischemic postconditioning on attenuating reperfusion injury [21, 34]. Thuny *et al*. showed reduction in the extent of myocardial edema by LPoC in STEMI [26]. However, results of many clinical trials evaluating LPoC [21, 22, 27, 30] and RPoC [23] have been controversial. In our meta-analysis, we combined positive [24, 26] and negative [21–23, 27, 30] studies and showed that LPoC and RPoC were associated with reduced myocardial edema after STEMI. However, the mechanisms underlying reduced myocardial edema by ischemic postconditioning need further investigation.

To address the effect of cardioprotective interventions on ischemia injury and myocardial edema, we selected T2 weighted cMRI, which is a water-sensitive technique that measures myocardial edema *in vivo* without using radiation or contrast agents and accurately represents the size of area at risk [35]. Our analysis showed that LPoC and RPoC decreased myocardial edema, but did not have any effect on the ischemia. The reasons for these effects are unclear. Myocardial edema includes intracellular and extracellular edema. However, cMRI could not distinguish between the two sources of extracellular edema, namely, intravascular water permeation and water release from necrotic cardiomyocytes into the infarcted area. Reperfusion-induced myocardial edema (extracellular space)increased wall thickness and stiffness favoring collagen deposition and fibrosis, which reduced expansion of the infracted area and left ventricular remodeling, regardless of myocardial salvage [36]. Moreover, recent studies using cMRI have demonstrated a bimodal pattern of myocardial edema after I/R injury, namely, an early phase that is reperfusion induced and occurs within 24 h and a late phase that represents the auto-healing process lasting at least 7days [37, 38]. However, the cMRI assessments were mainly performed within 1~7 days after PCI in the included trials. This suggested that ischemic postconditioning enhanced cardiomyocyte healing without affecting the infarction size. Previous studies have shown that LPoC and RPoC decreases inflammation and reactive oxygen species generation, which may prevent extracellular edema by increasing microvascular permeability [1, 39]. These studies partly explain the dissociation of the beneficial effects of ischemic postconditioning and structural damage in STEMI.

The main strength of our meta-analysis was that we assessed multiple structural parameterssuch as final IS, MSI, left ventricular volume, MVO, and myocardial edema in two settings of ischemic postconditioning namely, LPoC and RPoC using cMRIin STEMI patients. On the other hand, there were several limitations in our study. First, we included veryfew trials and studies and were unable to access individual patient data. Therefore,we may have underestimated the potential influence of comorbid conditions such as diabetes, dyslipidaemia, multi-vessel disease, and LAD as well as effects of cardiovascular medications such as β-blockers [40], glycoprotein IIb/IIIa inhibitors, and statins [10, 41]. Second, the relative small number of the enrolled subjects may have decreased the statistical power of our results. Third, we applied the random effect model based on I^2^ ≥ 50% and assumed normalized distribution [42]. But, we can't rule out heterogeneity influencing the outcomes of our study. Fourth, we excluded non–English language publications. Fifth, more studies are necessary to assess thec MRI data regarding the effect of ischemic postconditioning on cardiac structurein AMI with baseline TIMI flow grade 2~3, especially for RPoC. Finally, the long-term heart failure and cardiac mortality needs to be analyzed and the effect of therapy on cardiac structure needs to be confirmed in future clinical trials.

In conclusion, our meta-analysis of cMRI data showed that both LPoC and/or RPoC reduced the extent of MSI and myocardial edema in STEMI patients. However, there were no improvements in final IS, LV volume, and the incidence or the extent of MVO.

## MATERIALS AND METHODS

### Study search strategy and inclusion criteria

We performed this meta-analysis in accordance with PRISMA (Preferred Reporting Items for Systematic reviews and Meta-Analyses) [43]. We searched PubMed, EMBase, and Cochrane Library databases up to May 2017 with the following keywords:ischemic postconditioning,remote ischemic conditioning, ischemic postconditioning, acute myocardial infarction and percutanenous coronary intervention. Only prospective RCTs that were published in English and that reported STEMI undergoing percutanenous coronary intervention were included in this meta-analysis. Studies those (1) reported only cardiac enzyme levels and/or left ventricular ejection fraction; (2) did not use cMRI for structural assessment and (3) used pre-procedural TIMI flow grade ≥ 2 for potential spontaneous reperfusion [44] were excluded.

### Study selection, quality assessment and data extraction

Two investigators, Yadong Cui and Haiyang Gao, independently reviewed all abstracts and the full text according to the described search strategy and criteria. In case of disagreements, consensus was achieved by discussion. Quality assessment was performed according to the Jadad scoring system: randomization; blinding; withdrawals and dropouts. Each study obtained a score between 0 and 5 based on withdrawals and dropouts and those with a score ≥ 3 were considered high-quality [45].

Data extraction included trial design parameters such asyear, country,protocol algorithm, conditioning delay, symptom-to-balloon time, and follow up, and demographic data of patients such as age, gender, and presence or absence of diabetes mellitus, hypertension, smoking, dyslipidemia, stenting technique, multi-vessel, left anterior descending artery disease(LAD), and treatment with glycoprotein IIb/IIIa inhibitor, β-blockers, and statins.

### Evaluation of left ventricular structure by cMRI

We extracted endpoints assessed by structural cMRI imaging after PCI, which included primary endpoints such as final IS, MSI, and myocardial edema as well as additional endpoints such as LV volume and MVO. The final IS was assessed by late gadolinium enhancement of the cMRI images and expressed as percentage of LV mass [46]. The MSI was defined as the AAR minus IS; AAR was assessed by cMRI [47]. The myocardial edema was expressed as percentage of LV mass and evaluated by the T2 weighted method [33]. The LV volume was recorded as LV end-diastolic volume(LVEDV), or LVEDV index (LVEDVI), which is defined as LVEDV divided by body surface area [20]. The incidence and extent of MVO was expressed as percentage of LV mass and detected by late gadolinium enhancement of the cMRI images [20].

### Statistical analysis

Data was expressed as mean ± standard deviation or median ± interquartile range for continuous variables. We calculated WMD or SMD for LVR to obtain the pooled estimates with 95% confidence intervals (CIs). For dichotomous ones (reported with incidence), we calculated odds ratio (OR) with 95% CIs. We set I^2^ ≥ 50.0% as significant heterogeneity and used random-effects model for analyzing such parameters [48]. *P* < 0.05 (2-sided) was considered statistically significant. All statistical analysis was performed by Stata version 9.0(Stata Corporation, College Station, TX) and RevManversion 5.0(Cochrane Collaboration, Oxford, UK) softwares.
